# Modeling of Old Scars: Histopathological, Biochemical and Thermal Analysis of the Scar Tissue Maturation

**DOI:** 10.3390/biology10020136

**Published:** 2021-02-09

**Authors:** Alexey Fayzullin, Natalia Ignatieva, Olga Zakharkina, Mark Tokarev, Daniil Mudryak, Yana Khristidis, Maxim Balyasin, Alexandr Kurkov, Semyon Churbanov, Tatyana Dyuzheva, Peter Timashev, Anna Guller, Anatoly Shekhter

**Affiliations:** 1 Laboratory of Experimental Morphology, Institute for Regenerative Medicine, Sechenov First Moscow State Medical University (Sechenov University), 8-2 Trubetskaya st., Moscow 119991, Russia; fayzullin_a_l@staff.sechenov.ru (A.F.); b.maxim4432@yandex.ru (M.B.); kurkov_a_v@staff.sechenov.ru (A.K.); shekhter_a_b@staff.sechenov.ru (A.S.); 2 World-Class Research Center “Digital Biodesign and Personalized Healthcare”, Sechenov First Moscow State Medical University (Sechenov University), 8-2 Trubetskaya st., Moscow 119991, Russia; khristidis.yana@yandex.ru; 3 Chemistry Department, Lomonosov Moscow State University, Leninskiye Gory 1-3, Moscow 119991, Russia; nyu@kge.msu.ru (N.I.); timashev_p_s@staff.sechenov.ru (P.T.); 4 Institute of Photon Technologies, Federal Scientific Research Centre “Crystallography and Photonics” of Russian Academy of Sciences, 2 Pionerskaya st., Troitsk, Moscow 142190, Russia; olzakharkina@gmail.com (O.Z.); churbanov.semyon@gmail.com (S.C.); 5 Sklifosovsky Institute for Clinical Medicine, Sechenov First Moscow State Medical University (Sechenov University), 8-2 Trubetskaya st., Moscow 119991, Russia; tokmv9@gmail.com (M.T.); mdl.surg@gmail.com (D.M.); dtg679@gmail.com (T.D.); 6 Department of Advanced Biomaterials, Institute for Regenerative Medicine, Sechenov First Moscow State Medical University (Sechenov University), 8-2 Trubetskaya st., Moscow 119991, Russia; 7 Laboratory of Clinical Smart Nanotechnologies, Institute for Regenerative Medicine, Sechenov First Moscow State Medical University (Sechenov University), 8-2 Trubetskaya st., Moscow 119991, Russia; 8 Department of Polymers and Composites, N. N. Semenov Institute of Chemical Physics, 4 Kosygin st., Moscow 119991, Russia; 9 ARC Centre of Excellence for Nanoscale Biophotonics, Graduate School of Biomedical Engineering, University of New South Wales, Kensington, NSW 2052, Australia

**Keywords:** scarring, hypertrophic scar, animal model, scar maturation, quantitative histopathology, collagen

## Abstract

**Simple Summary:**

Severe skin scars (i.e., hypertrophic and keloid) induce physical and emotional discomfort and functional disorders such as contractures and body part deformations. Scar’s response to treatment depends on “maturity”, which increases with time but is not merely proportional to it. When “fresh”, scars are relatively more treatable by conservative methods, while the treatment is only partially efficient. In contrast, surgery is a preferred approach for the older scars, but it is associated with a risk of the scar regrowth and worsening after excision if unrecognized immature scar tissue remains in the operated lesion. Therefore, to develop better treatment and diagnostics of scars, understanding of the scar maturation is essential. This requires biologically accurate experimental models of skin scarring. The current models only mimic the early stages of skin scar development. They are useful for testing new scar-preventing approaches while not addressing the problem of the older scars that exist for years. In our study, we demonstrate a new rabbit model of “old” scars and explore what happens to the scar tissue during maturation. We define measurable signs to delineate the scar development stages and discuss how this knowledge can improve scar diagnostics and treatment.

**Abstract:**

Mature hypertrophic scars (HSs) remain a challenging clinical problem, particularly due to the absence of biologically relevant experimental models as a standard rabbit ear HS model only reflects an early stage of scarring. The current study aims to adapt this animal model for simulation of mature HS by validating the time of the scar stabilization using qualitative and quantitative criteria. The full-thickness skin and perichondrium excision wounds were created on the ventral side of the rabbit ears. The tissue samples were studied on post-operation days (PODs) 30, 60, 90 and 120. The histopathological examination and morphometry were applied in parallel with biochemical analysis of protein and glycosaminoglycans (GAGs) content and amino acid composition. The supramolecular organization of collagen was explored by differential scanning calorimetry. Four stages of the rabbit ear HS maturation were delineated and attributed with the histolomorphometrical and physicochemical parameters of the tissue. The experimental scars formed in 30 days but stabilized structurally and biochemically only on POD 90–120. This evidence-based model can be used for the studies and testing of new treatments of the mature HSs.

## 1. Introduction

Hypertrophic scars (HSs) are benign foci of skin fibrosis occurring following traumas, burns and surgical operations and, in contrast to the keloids, remaining within the borders of the original wound [1,2]. These scars are common in clinical practice [3] as they affect 4.5–16% of the population [4]. HSs are elevated above the healthy skin, have reduced elasticity, and may result in the formation of scar contractures. Often, they differ in color from the skin, pruritic or painful. HS may result in disability and social isolation [3].

HSs are dynamic structures. After the wound epithelization, the granulation tissue (and, in special cases such as surgically treated burns, other tissues of the wound bed [5]) transforms into scar tissue [6,7]. This process may take months. Then, the scar tissue undergoes remodeling signifying its adaptation to the local biomechanics and systemic response to the injury. The changes of the external appearance, histological structure and functional activity of the scar reflect the scar maturation. They include growth and consequent decrease of the scar volume and the turgor, color changes (towards more similar to the skin), and sometimes the variations of the intensity of the subjective sensations (itching, pain, etc.) [1,8,9]. According to the literature, the maturation of HS in humans may take from three to six months [10] or up to several years [11]. It is recommended to consider a 6–12 month period as an average time needed for an HS maturation [12].

The morphological substrate of the scar maturation is not fully known [13]. The scar time-course is associated with the reorganization of the extracellular matrix, downregulation of inflammatory reactions, decrease in vascularity and phenotype modification of the fibroblasts, including the fibroblasts-myofibroblasts transitions serving the scar contraction [1,11,13,14,15,16,17,18]. The speed of the maturation of the scar depends on the delay of the epithelization, the intensity of the contraction [18,19,20], as well as the size and the location of the scar (the larger and deeper wounds have higher chances for prolonged maturation).

Importantly for clinical practice, the mature scar tissue becomes functionally inert and much less responsive to therapies than the scars existing for less than one year [3]. Then, mature scars represent a therapeutically unanswered challenge and are usually considered for surgical revision [9,12]. At the same time, the surgical interventions in young scars (when the HS and keloids are less clearly differentiated [21]) are associated with the risk of excessive scarring after the excision [1,18]. Therefore, a clearer understanding of the HS maturation process is needed.

Animal models of HSs are relatively limited due to the differences in skin and subcutaneous tissue structure and other physiological aspects between humans and animals [22]. The key distinction is a higher elasticity and displacement ability of the animal skin resulting in faster wound closure in animals [23]. This is considered an important limitation in the modeling of HSs. A breakthrough model of HS was proposed by T. Mustoe and co-authors [20], who found that full-thickness ischemic wounds of the ventral side of the rabbit ears can heal with the formation of scars visually similar to the human HS. This model [20] relies on the four-six circular excisions of the skin and subdermal auricular perichondrium on the ventral side of the rabbit ears. The excisions are performed by a 6–7 mm biopsy puncher under a dissection microscope. In this model, epithelization occurred in 15–20 days and followed by the formation of scar tissue in 30–60 days with the maximum elevation of the scar at ~30 days after the operation. The model [20] was validated in more than 70 studies as a reproducible tool for the investigation of new diagnostic modalities and topical anti-scarring treatments [23,24,25]. The majority of these works were performed when the scars had the maximum height. This implies that the scar maturation was not completed yet. At the same time, the arsenal of the tools to objectively define the degree of scar maturity is limited [13,21]. Moreover, the limited duration of scar models, including the conventional one in rabbit ear, remains a serious obstacle for the extrapolation of the in vivo experimental finding to the humans [22].

Here, we adapted the rabbit ear HS model [20] for the simulation of mature HSs that can be observed in a reasonable time (4 months) and used for the development of the treatment strategies applicable to the old scars. To define the scar stabilization time point, we qualitatively and quantitatively defined the histological and physicochemical patterns of the experimental HS maturation.

## 2. Materials and Methods

### 2.1. Surgical Procedures

The experiment in eight chinchilla rabbits (males, 2–2.5 kg) was approved by the Local Ethical Committee of Sechenov University. The animals were kept one per cage and provided with complex granulated laboratory chow and constant access to water. The HS model was established on the left rabbit ears following the published protocol [19,20], with minor modifications. Briefly, the full-thickness skin and perichondrium defects were created using a 10 mm biopsy puncher. For the surgery, the animals were anesthetized by intramuscular injection of Zoletil 100 (Virbac, Carros, France; 6 mg/per 1 kg of animal body weight) with local infiltration of the operating field with Novocain 0.5%. The wounds were dressed with Cosmopor^®^ E patches (Paul Hartmann, Heidenheim an der Brenz, Germany). Postoperative antibacterial therapy was done by intramuscular injections of Baytril 5% (Bayer, Germany; 5 mg of Enrofloxacin per 1 kg of animal body weight) daily for five days after surgery.

On the 30th, 60th, 90th and 120th postoperative days (POD), the rabbits were euthanized by the injection of Zoletil 100 (60 mg/kg of animal body weight). Two animals per time point were sacrificed. Three scars per animal were harvested (together with 2–3 mm rim of surrounding skin) and used in further studies. Each of the tissue samples was divided into three parts: a half of each sample was fixed in 10% neutral-buffered formalin; a third of the original sample was immersed in an OCT cryogel and snap-frozen in liquid nitrogen for the immunohistochemical staining. The remaining fragment of each scar without skin rim was placed in saline for further biochemical assays. The matching intact (control) skin fragments were dissected from the right ears of the animals that were sacrificed on POD 30.

### 2.2. Histology

Four-μm-thick sections of the formalin-fixed-paraffin-embedded tissue samples were stained with hematoxylin and eosin (H&E) and with Picrosirius red (PSR) and examined using a Leica DM4000 B LED microscope, equipped with a Leica DFC7000 T digital camera running under the LAS V4.8 software (Leica Microsystems, Heerbrugg, Switzerland). The specimens were studied under conventional (H&E, PSR) and polarized light (PSR).

### 2.3. Immunohistochemistry (IHC)

Four-μm-thick frozen tissue sections were prepared using a Leica CM1950 cryotome, treated with 3% H_2_O_2_ and background block (Cell Marque, Germany), incubated with mouse monoclonal primary antibodies against collagen type I (GTX26308, GeneTex, Irvine, CA, USA, diluted 1:4000) and HRP-conjugated secondary goat antibodies (G-21040, Invitrogen, Carlsbad, CA, USA, diluted 1:1000). The formed immune complexes were revealed by DAB with hematoxylin contrasting.

### 2.4. Morphometry

The scar thickness was measured in each sample at five sites as a distance from the interior surface of the epithelium to the upper surface of the cartilage plate. The fibrotic index was evaluated as a ratio (%) of the thickness of scar tissue with parallel-oriented bundles of collagen to the mean entire scar thickness. The number of cells in the scar tissue was counted at the 400× magnification in 10 random fields of view in each sample, and the mean number of cells per one mm^2^ of the tissue section was calculated. The histological signs of inflammation in the scar vs. the surrounding normal skin were evaluated using semiquantitative scoring (Table 1).

### 2.5. Chemical Analysis

Dried tissue specimens were hydrolyzed, and amino acid analysis was done with a Hitachi-835 amino acid analyzer (Hitachi Co., Tokyo, Japan) in the standard way for protein hydrolysate analysis with cation-exchange separation and ninhydrin post-column derivatization. The collagen content was estimated, assuming that the hydroxyproline (Hyp) content in collagen is 13.5% [26]. For the glycosaminoglycan (GAG) assays, small tissue pieces were dried, weighed, and immersed in a water bath at 85 °C for 12–15 min for the collagen triple helix denaturation. Thereafter the pieces were digested in 1 mg/mL trypsin solution containing 0.02% (*w*/*v*) sodium azide for 24 h at 37 °C; the suspension was centrifuged. The GAGs were quantified in the supernatant by 1,9-dimethylmethylene blue assay (DMMB, MP Biomedicals) using the published method [27] with minor modifications. When preparing the stock solution, we replaced HCl with CH_3_COOH, excluded the addition of NaCl, and added the treatment of the solution in an ultrasonic bath for 5 min. These changes allowed substantial improvement of the calibration plot. Calibration standards were prepared using chondroitin sulfate A (C6737, Merck) solution. Absorbance at 589 nm was measured with a UV-1800 spectrophotometer (Shimadzu Co., Kyoto, Japan) using a DMMB solution as a reference.

### 2.6. Thermal Analysis

Tissue samples of approximately 7–10 mg were blotted with tissue paper to remove surface water and placed in hermetically sealed aluminum pans. Differential scanning calorimetry (DSC) measurements were performed using a Phoenix DSC 204 calorimeter (Netzsch, Selb, Germany). Heating was carried out from 20 °C to 90 °C at the scanning rate of 10 °C/min. The resulting DSC curves were analyzed using Proteus^®^ Thermal Analysis software. The heat of collagen denaturation was normalized to the dry weight of the tissue. Deconvolution of DSC data in the 40–85 °C region into Gaussian peaks was performed by multi-peaks fitting using Origin 8.0 software. The fraction of the corresponding collagen population in the mixture was estimated via the area under each peak (estimated by peak deconvolution).

### 2.7. Statistical Analysis

The statistical analysis was performed with standard program packages GraphPad Prism 8.00 for Windows (GraphPad Software, Inc., San Diego, CA, USA) and SPSS 21.0 statistical software (IBM, Armonk, NY, USA). The distribution of the quantitative data was checked by Shapiro–Wilk’s normality test. The intergroup differences of the normally distributed data were analyzed by the one-way ANOVA followed by Tukey’s multiple comparison test. The differences in the histological scores were evaluated using Kruskal–Wallis test followed by Dunn’s multiple comparison test. Nonparametric correlations were analyzed to examine associations between the studied variables. Two-tailed statistical tests were used. *p*-values ≤ of 0.05 were considered statistically significant. The statistical analysis results were depicted as scatter plot graphs of the mean values (for quantitative data) or median values (for inflammation index) and standard deviations of the mean (SD).

## 3. Results

### 3.1. Morphometric Analysis, Histology, and Immunohistochemistry

**Figure 1:**
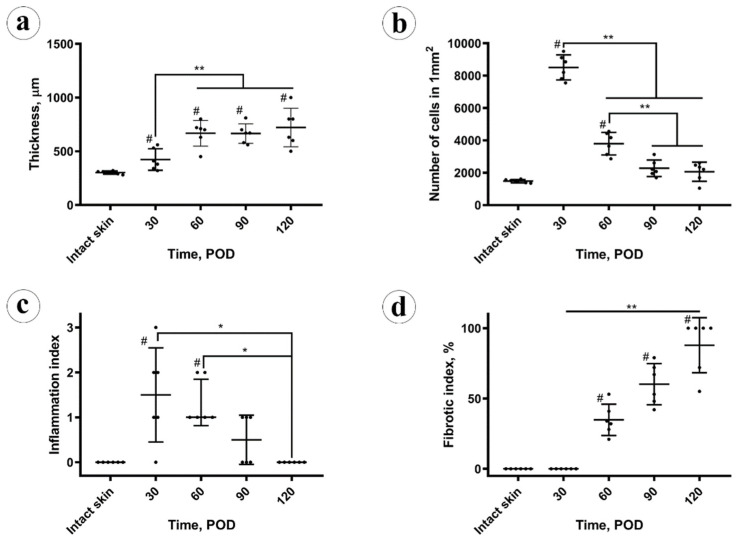
Morphometry analysis. (**a**) Thickness of the dermal parts of the intact skin and experimental scars; (**b**) cellular density; (**c**) inflammation index; (**d**) fibrotic index. Statistical significance: * *p* ≤ 0.05, ** *p* ≤ 0.01, # shows *p* ≤ 0.05 in comparison to control.

The histomorphometric analysis results are presented in Figure 1. The dynamics of scar thickness is depicted in Figure 1a. On POD 30, the scars were thicker than the intact skin (423 ± 100 µm; with 95% confidential interval for the mean (CI 95%) (319, 528) µm vs. 302 ± 15 µm; CI 95% (286, 317) µm); the difference was statistically significant (*p* = 0.014). On POD 60, the scar thickness increased significantly (*p* < 0.001), in comparison with the POD 30, and reached the elevation of 668 ± 120 µm; CI 95% (543, 794) µm. After this time point, there were no statistically significant changes of scar thickness, which was 665 ± 90 µm on POD 90 (CI 95% (570, 760) µm) and 722 ± 180 µm on POD 120 (CI 95% (533, 911) µm).

The cellular density (Figure 1b) peaked at POD 30, then decreased until POD 90 and stabilized between PODs 90 and 120. The changes in cellularity of the scar tissue were statistically significant only between the POD 30, POD 60 and POD90 time points. There was a statistically significant moderate negative correlation (Pearson’s correlation coefficient, Rs = −0.494; *p* = 0.014) between scar thickness and cellular density.

The inflammation index in scars (Figure 1c) on POD 30 was higher than in the intact skin (statistically significant difference, *p* = 0.007). Later, between POD 30 and POD 60, statistically, it did not change (*p* = 0.727), and then it decreased from POD 60 to POD 90 (*p* = 0.030) and stabilized between POD 90 and 120 (*p* = 0.56). There was no statistically significant correlation between the scar thickness and the inflammation index, while the cellularity and inflammation index were mutually positively correlated (Rs = 0.586, *p* = 0.003).

The fibrotic index of the scars (Figure 1d) increased from POD 30 (zero) to POD 60 (33 ± 10%; CI 95% [23, 44]%) and POD 90 (60 ± 16%; CI 95% [44, 76]%) and then reached a maximum on POD 120 (87 ± 22%; CI 95% [64, 100]%). The fibrotic index positively correlated with the scar thickness (Rs = 0.478, *p* = 0.018). It also was strongly negatively correlated with the cellularity of the scars (Rs = −0.826, *p* < 0.001) and moderately negatively correlated with the inflammation index (Rs = −0.661; *p* < 0.001).

**Figure 2:**
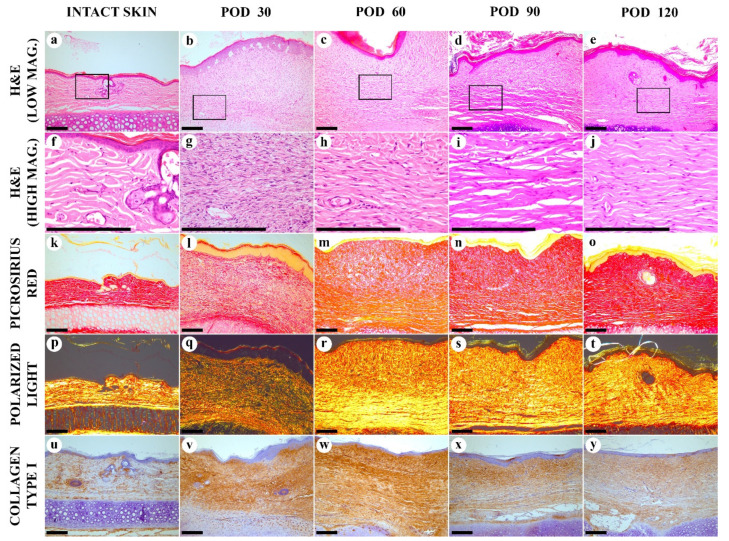
Histological examination of the intact skin and experimental scars. (**a**–**j**) hematoxylin and eosin (H&E) staining; (**k**–**t**) Picrosirius red (PSR) staining; (**u**–**y**) immunohistochemistry (IHC) staining for collagen type I. Columns depict the studied groups (intact skin and experimental scars on PODs 30, 60. 90 and 120). Images were taken in bright-field (**a**–**o**,**u**–**y**) or polarized light (**p**–**t**) illumination. Scale bars are 200 µm.

The histological structure of the experimental scars was notably different from those of the intact skin (Figure 2).

On POD 30, the scar was immature and formed by the fibrosing granulation tissue (Figure 2b,g). The spindle-shaped fibroblasts were predominating, with a few mitoses visible. The cellular density in this tissue was much higher than in the intact derma, comprising (8.5 ± 0.1) × 10^3^ vs. (1.5 ± 0.8) × 10^3^ cells per mm^2^ of the tissue section, respectively (*p* < 0.001). The tissue was highly vascularized and contained capillaries, arterioles, and venules. Minor diffuse and perivascular infiltration by macrophages, lymphocytes and, rarely, neutrophils was notable. The deep parts of the scar contained fewer cells and had slightly more organized collagen bundles, while the upper part (underlying the epidermis) demonstrated the fine, randomly oriented fibroblasts and interwoven collagen fascicles. The matrix stained red by PSR (Figure 2i), but the staining intensity was weaker than in the intact skin dermis. Diffuse green color dominated in PSR-stained scar samples visualized by the polarized light microscopy, indicating immaturity of collagen (Figure 2q). Immunohistochemical staining for collagen I, on POD 30, show moderately intense, diffuse, and uneven staining of the entire scar tissue (Figure 2v). The intensity of staining was higher than in the intact skin (Figure 2u).

On POD 60, scar tissue became more mature in comparison with POD 30. The number of fibroblasts was significantly reduced, the mitoses were not found, and the young cells with large cytoplasm were almost absent. The vascularity decreased significantly, as well as the cellular inflammatory infiltration. Only a few perivascular infiltrates containing lymphocytes and macrophages were revealed. The upper and the bottom layers of the scar tissue with random and parallel collagen alignment, respectively, became more clearly differentiated. This distinction was mostly visible on PSR-stained samples. The upper layer was less dense and stained red by PSR, while the bottom layer demonstrated yellow-red staining. By polarized light microscopy, the PSR-stained samples revealed a green-to-yellow shift, comparing with POD 30, with the bright yellow staining in the bottom part and dim yellow in the upper part of the scar. Immunohistochemistry reaction revealed diffuse, strong expression of collagen type I over the scar; the staining intensity on POD 60 was higher than on POD 30. Moreover, a mild reparative reaction in the cartilage plate emerged. This included hypertrophy of chondrocytes and partial replacement of the intact auricular cartilage with the fibrous cartilage tissue.

On POD 90, in H&E stained samples, the mature fibrous connective tissue with thick, parallel-oriented collagen bundles located at the bottom part of the lesion formed almost half of the scar. In the upper layer of the scar, collagen fascicles became thicker and more aligned than on POD 60. The total cell number, vascularity and inflammation were moderately reduced in comparison to the same characteristics on POD 60. When stained by PSR, the whole scar block had a more homogenous red staining. Polarized light microscopy of PSR-stained samples showed the bright yellow luminescence in both layers of the scar tissue. The intensity of IHC staining for collagen I reduced in comparison with the observation of PODs 30 and 60.

On POD 120, the scar structure became relatively homogenous. The collagen bundles aligned parallel to the epidermal surface. The average thickness of the bundles was reduced, compared with the previous time point. Diffuse-positive IHC staining for collagen I was observed together with homogenous red staining by PSR under bright field illumination and homogenous yellow PSR luminescence under polarized light. The cellular composition and the vascular patterns were the same as on POD 90. The inflammation signs decreased and approached the level of the intact skin derma.

### 3.2. Chemical Analysis

**Figure 3:**
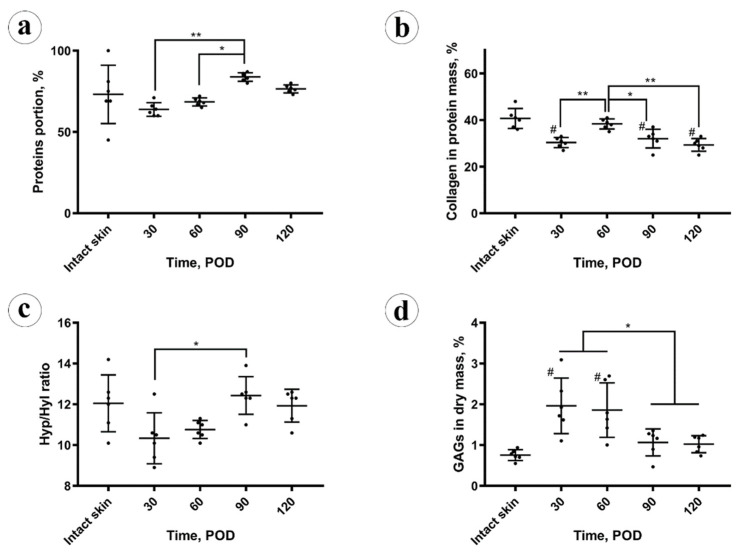
Chemical analysis: (**a**) protein amount; (**b**) collagen amount in the protein mass; (**c**) amino acid analysis; (**d**) spectrophotometry of glycosaminoglycans (GAGs). Statistical significance: * *p* ≤ 0.05, ** *p* ≤ 0.01. Mean values ± SD; # shows *p* ≤ 0.05 in comparison to control.

The total protein content (Figure 3a) in the intact skin was 73 ± 18% (CI 95% (54, 92)%). In the scars, on POD 30 it was slightly reduced (not at the level of statistical significance, *p* = 0.103), comparing to the intact skin 64 ± 4% (CI 95% (59, 68)%). It gradually, not statistically significantly (*p* = 0.087), increased to 69 ± 2% (CI 95% (66, 71)%) on POD 60. Next, on POD 90, the protein amount grew up to 84 ± 3% (CI 95% (81, 87)%), overcoming the values previously observed in the scars and skin (*p* < 0.001). On POD 120, it statistically significantly decreased (*p* = 0.003), comparing to POD 90, and measured 77 ± 2% (CI 95% (74, 79)%), approaching the value observed in the intact skin. The total protein content in the experimental scars positively correlated with the scar thickness (Rs = 0.566, *p* = 0.004) and the fibrotic index (Rs = 0.738, *p* < 0.001), while was strongly negatively associated with the cellularity (Rs = −0.747, *p* < 0.001) and inflammation (Rs = −0.520, *p* = 0.009).

The collagen content (Figure 3b) in the protein mass of the scars on POD 30 was significantly (*p* < 0.001) lower than in the intact skin (30 ± 3%; CI 95% (28, 33)% vs. 41 ± 4%; CI 95% (36, 45)%, respectively). On POD 60, collagen amount in scars increased to the skin-matching level 38 ± 2% (CI 95% (36, 41)%) and then gradually decreased to 32 ± 4% (CI 95% (28, 36)%) on POD 90 and 29 ± 3% (CI 95% (27, 32)%) on POD 120, finally dropping below the intact skin measurements. The amount of collagen in the protein mass of the scars did not correlate with the scar thickness, fibrotic index, cellularity, inflammation, and total protein content.

Amino acid analysis results are demonstrated in Figure 3c. A statistically significant decrease in the molar hydroxyproline to hydroxylysine (Hyp/Hyl) ratio in the scars (10 ± 1%) vs. intact skin (12 ± 1%) was found on POD 30 (*p* = 0.048) and on POD 60 (11 ± 1%; *p* = 0.044). This ratio returned to the normal skin values on POD 90 and POD 120 (12 ± 1%). The Hyp/Hyp ratio in the scars positively correlated with the total amount of protein (Rs = 0.596, *p* = 0.002) and fibrotic index (Rs = 0.606, *p* = 0.02) and negatively with the cellularity (Rs = −0.509, *p* = 0.011). There were no statistically significant correlations between the Hyp/Hyl ratio and scar thickness, inflammation, and collagen content.

The amount of sulfated GAGs in the scar dry mass (depicted in Figure 3d) was sharply and statistically significantly (*p* = 0.002) increased in the scars on POD 30 (2.0 ± 0.7%), in comparison with the intact skin (0.8 ± 0.1%). On POD 60 it equaled 1.9 ± 0.7% (no statistical difference vs. POD 30), and then decreased to 1.1 ± 0.3% on POD 90 and 1.0 ± 0.1% on POD 120. The drop of GAGs content between POD 60 and POD 90 was statistically significant (*p* = 0.027), while later the concentration of GAGs was statistically unchanged. The GAGs amount positively correlated with the cellularity (Rs = 0.698, *p* < 0.001) and the inflammation (Rs = 0.543, *p* = 0.06). Negative correlations were observed between the GAGs content and the thickness of scars (Rs = −0.428, *p* = 0.037), fibrotic index (Rs = −0.626, *p* = 0.001), total protein amount (Rs = −0.597, *p* = 0.002), and Hyp/Hyl ratio (Rs = −0.509, *p* = 0.011).

### 3.3. Thermal Analysis

**Figure 4:**
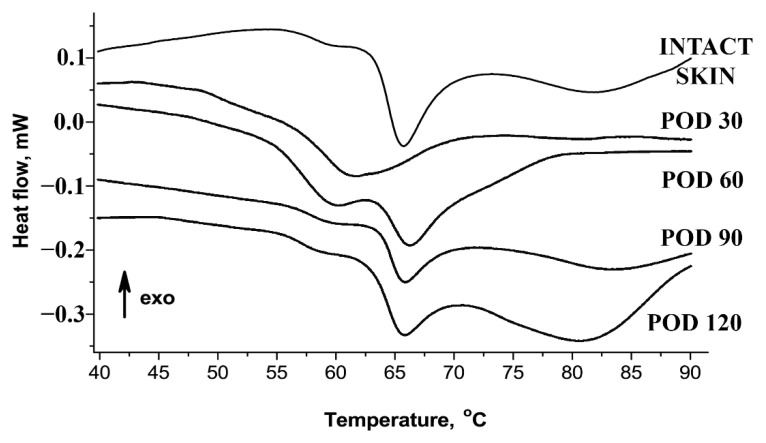
Thermography of skin and scar tissues.

Figure 4 and Table 2 show the results of thermal analysis of collagen supramolecular structure. The denaturation endotherm of intact skin dermis could be decomposed into two separate thermal transitions (collagen populations). The first, the low-temperature transition (Tp1), was visible at ~59 °C. It represented ~14% of the total calorimetric enthalpy and attributed to the denaturation of the recently synthesized collagen characterized by immature and weak crosslinking profile [28,29]. The main transition (Tp2, peak at ~66 °C) represented more than 60% of the total enthalpy and corresponded to the denaturation of the mature collagen population stabilized by the crosslinks. In the scars, on POD 30, the bulk of collagen was represented by a single fraction with the reduced amount of crosslinks reflected in a single peak at ~61 °C. Then, the conditionally defined ratio of “immature” collagen in the scars on POD 30 significantly increased and reached approximately 50%. On POD 60, two fractions of collagen became detectable in the scars, including the Tp1 for the low-crosslinked collagen and the Tp2 for the normal one. The ratio of “immature” collagen on POD 60 overcame those of the intact dermis (~35%). On PODs 90 and 120, the endotherms of collagen denaturation were similar to the curves observed in the intact skin.

## 4. Discussion

In this study, the process of HS maturation was modeled and systematically explored.

Conventionally, the rabbit ear HS model is used for the evaluation of various treatments targeting the process of scar formation [19]. The treatment usually starts on POD 30 and continues for the next four weeks [4,7,14,15,16,17,30,31,32,33,34,35,36]. The results of the current study show that during this period, the scar is not stable yet. This implies that many treatment effects reported in the respective literature occur on the background of the natural scar maturation. Whether the proposed treatment is equally efficient in the old, mature HSs remains an open question.

Here, we extended the period of observation of rabbit ear HSs up to POD 120. Only a few brief reports have been published before our work on the structure of the rabbit ear HS scars existing longer than two months since experimental wounding [20,37]. Previously, to create such long-lasting scars, the large (1.5–2 cm × 4.5–7 cm) full-thickness excisional/electrocauterized wounds were made on the ventral side of rabbit ears instead of punch biopsies, and the histological data on these scars was fragmentary [20]. The authors reported the elevation of the scar above the skin level, organization of fibrosis, mild inflammatory signs in the scar tissue and thickening of the subdermal cartilage during the period to POD 90 (without specific staging), while the macroscopic observations continued for approximately nine months. The large size of the wounds and the alternative surgical technique made this chronic scarring model less reproducible than the classical approach; the further usage of this chronic HS model was minimal. In the present work, we challenged ourselves to reconcile the need in a mature HS model with the reproducible surgical methodology and rational observation time (4 months). We achieved this by a minor increase of the punch biopsy wounds diameter (from 7 to 10 cm). By the application of the quantitative and semiquantitative methods for the objective evaluation of the scars, we identified four stages of the rabbit ear experimental HS maturation.

*Stage 1, the early immature scar.* On POD 30, the scar tissue was immature. It consisted of the densely vascularized organizing granulation tissue with very high cellularity and increased inflammation. Biochemical signature included the reduced total protein and collagen contents, decreased Hyp/Hyl ratio, and sharply enlarged amount of GAGs. The scar was thicker than the intact skin, while there were no organized fibrous collagen bundles with parallel alignment yet, and the polarization light luminescence of collagen stained with PSR was dim and diffuse. Thermal analysis revealed the loss of the two skin-specific thermal transitions on the DSC curve and the formation of the single peak at ~61 °C; that corresponded to the large fraction (~50%) of immature collagen.

*Stage 2, the late immature scar.* Between PODs 30 to 60, the scars underwent intensive changes. The thickness of the scar increased, while the cellularity and the inflammation index reduced. The total protein content started to rise, while the collagen amount was restored at the level of the intact skin. There was an increase of the fibrotic index. A gradual formation of the layered scar structure was visible by polarization microscopy. The IHC expression of collagen type I was relatively increased across the whole scar. The Hyp/Hyl ratio was lower than in the skin. The fraction of immature collagen (by DSC) was still much higher than in the skin. This can be explained by the low activity of lysyl oxidase at the early stages of fibrosis of granulation tissue [38], resulting in the reduced amount of crosslinked collagen in 4–10 times [23]. The amount of GAG still was very high.

*Stage 3, the early mature scar.* Between PODs 60 to 90, the scar thickness stabilized, the cellularity decreased, and the inflammation almost disappeared. Two layers of the scar tissue became visible by H&E, while the polarization microscopy revealed an almost homogenous bright luminescence in PRS-stained samples. The fibrotic index continued to grow. There was a notable change of the collagen bundles alignment to a more parallel one that was previously defined as one of the signatures of HS [39]. The total protein content grew, and the amount of GAGs and collagen type I expression continued decreasing. Hyp/Hyl ratio and the DSC signature approached the skin values. The observed dynamics of GAGs content corresponds to the previously reported data on the concentration of chondroitin-sulfate in the granulation tissue and immature HSs (six times higher than in the skin) and mature scars (slightly higher than in the skin) [6]. The changes in the Hyp/Hyl ratio revealed during the 90 days post-wounding could be attributed both to a shift in the ratio of collagen types [40] and a change in the activity of prolyl and lysyl hydroxylases and lysyl oxidase [38,41]. In the current study, the Hyp/Hyl ratio in scars mostly remained close to the values of normal skin [42] and dropped below the “skin range” only in the first two stages of scar maturation (up to POD 60). This reduction in Hyp/Hyl ratio may be associated with a possible increase of the relative amount of collagen type IV due to a high content of vascular elements (the commonly known localization of this collagen type) as it is characterized by a low Hyp/Hyl value (2.6–3.0) [42]. It is likely that the Hyp/Hyl ratio grew during stage 3 of scar maturation (PODs 60–90) due to the temporary increase of the amount of collagen type III, which has a high Hyp/Hyl value (22.0) [42].

*Stage 4, the late mature scar.* On PODs 90 to120, the scar thickness was stable, and the cellularity almost did not change, as well as the inflammation index, while the fibrotic index was increasing. The upper and bottom layers of the scar tissue were not further discernible histologically. The organization and alignment of collagen bundles are enhanced. Collagen I expression slightly reduced, comparing the earlier stages of scarring. Moreover, the total protein collagen content diminished, while the Hyp/Hyl ratio did not change much. GAGs amount and the immature collagen fraction (defined by DSC) remained unchanged. This corresponds with the 2–2.5 times increase of lysyl oxidase in 3-month-old human postburn scars compared to normal skin as reported earlier [38]. The restoration of the normal skin Hyp/Hyl ratio in scar tissues observed by POD 120 may indicate the re-balancing of the production of key macromolecules that determine the formation of collagen fibers.

We propose several interpretations of correlation analysis results relevant to the mechanisms of scarring and the possible implications for further HS modeling and treatment.

First, we found that the thickness of the scar was continuing to grow between POD 30 and 60, while the peak value of cellular density was achieved on POD 30 and decreased later. This indicates that cellular hyperplasia was not responsible for the scar volume increase at least after POD 30. Moreover, the absolute values of the Rs indicated that the thickness of the scars was more dependent on the extracellular matrix changes (fibrotic index) rather than on the fibroblast cellular dynamics and inflammation. At the same time, the fibrotic index strongly negatively correlated with the cellularity and inflammation index.

Next, the most notable increase in the total protein content was observed during stage 3 of scar maturation (POD 60 to 90). Then, some decrease in protein concentration was detected. This corresponded with the organization of the two-layered structure with thick collagen bundles followed by its remodeling into a more homogenous and regular collagen framework with thinner bundles that may be attributed to the activity of metalloproteinases. Total protein content, scar thickness and the fibrotic index correlated positively with each other and negatively with the cell density. This means that the increased protein amounts, the scar volume, and the fibrosis were supported not by the fibroblast hyperplasia (the increase in numbers) but by the hypertrophy of the cells (enhanced functional efficacy, which is usually associated with enlarged dimensions of the cells). It seems rational to suggest that the cytostatic and anti-inflammatory treatment of the scars may result in an increase of the total protein content or the acceleration of the organization of fibrosis, contributing to the overall scar maturation. Interestingly, the collagen content did not correlate with the scar thickness, fibrotic index, cellularity, inflammation, and total protein content. This may testify to the role of non-collagenous compounds, possibly of proteinaceous nature (e.g., proteoglycans, glycoproteins, or enzymes), in the formation of the scar volume.

Finally, a sharp increase in the content of sulfated GAGs positively correlated with cellularity and inflammation and negatively associated with the scar thickness, fibrotic index, and total protein amount. This indicates that GAGs are unlikely to be the non-collagenous extracellular matrix component responsible for the scar prominence. Importantly, the accumulation of GAGs and protein in the scars was separated in time. The GAGs were increased up to POD 60, whereas after this time point, GAGs concentration reduced, while the protein accumulation grew dramatically. This corresponds with the previously shown accumulation of sulfated GAGs in granulation tissue [6] and in immature HSs [43]. It seems reasonable to suggest that GAGs as highly polar water-binding compounds work in the immature scars for the mechanical stress distribution over an increased volume when the structural components of the scar are not sufficient to bear the tension. This is similar to the “shock absorbers” function of GAGs described, first of all, in cartilage [44]. The molecular mechanisms behind the apparent switch from GAGs to protein accumulation in the maturating HS are not clear yet. Different rates of GAG and protein synthesis and transformations are thought to be among the factors that can contribute to this. However, the estimation of the rates and energy costs of these processes is complicated, as numerous conditions can affect them [45,46].

We think that these findings provide the rationale for the stage-specific scar-reduction therapeutic strategies. For example, GAGs potentially can be targeted at stages 1 and 2 of scar maturation by physical therapies that promote the redistribution of water bound by GAGs and activate the mechanosensory responses of cells [47]. Alternatively, various pharmacological strategies can be applied to control GAGs metabolism or local concentration [46,48]. Despite some potentially positive changes such as an increase of the elastic fibers content or clinical improvement was observed after the treatment of early HS with chondroitinases in a conventional rabbit ear model [49] and in human keloids [50], respectively, the GAG-reducing approach requires certain caution and further studies as it may result in significant changes of the biomechanical and humoral signaling balance following the release of the growth factors from the GAG-associated depot and redistribution of the mechanical tensions due to displacement of unbound water [44,51]. Moreover, recent data show that highly sulfated chondroitin sulfate may protect fibroblasts of the HS from activation of alpha-smooth actin expression after stimulation by transforming growth factor-beta, the major regulator of fibrosis [52]. In this study, we did not identify the individual GAG types as this question has been addressed in several studies [6,43,52,53,54,55,56,57,58,59,60,61,62,63,64,65,66]. However, our current findings clearly point to the role of GAGs in HS maturation, and therefore we expect that further, more targeted research in this area may help for better control of the scar tissue remodeling.

On the other hand, the correction of the protein composition and turnover, for example, via the biological approaches such as the control of cellular phenotype and secretion profiles [67] or ECM remodeling [68,69], looks more justified at the later stages of scarring, which are more relevant to the tasks of the reconstructive and plastic surgery. Considering our findings point to the role of non-collagenous protein components of ECM in the increase of the thickness, it looks especially promising to explore the scar matrisome components beyond the collagens. With this regard, the -omics technologies enhanced with data science methods [70,71] may play a key role in the identification of new targets for scarring control in the ECM milieu.

## 5. Conclusions

We conclude that the mature HSs can be reproducibly modeled in rabbit ears with a minor modification of the surgical technique vs. the conventional approach. The results of the study show that at least three months are needed for the rabbit ear HS maturation. The described objective criteria of the four HS maturation stages will contribute to the better experimental design of the studies of the new diagnostic and treatment methods of the established human skin scars.

## Figures and Tables

**Table 1:** Criteria of semiquantitative histological scoring of the scar tissue.

Inflammation Index	Criteria
0	Similar to intact dermis
1	A few focal inflammatory cell infiltrates
2	Multiple focal inflammatory cell infiltrates or/and microcirculatory disorders in single blood vessels
3	Diffuse inflammatory cell infiltration or/and microcirculatory disorders in the majority of blood vessels

**Table 2:** Temperature picks (Tp) and low-temperature mass portions of thermographs of studied tissues (statistical significance in comparison with the intact tissue: * *p* ≤ 0.05).

Samples	Mean ± St. Deviation		
	Tp1, °C	Tp2, °C	The Low-Temperature Peak Ratio, %
Intact Skin	58.9 ± 1.1	65.6 ± 0.9	14.2 ± 5.5
Scars, POD 30	61.6 ± 1.4		49.9 ± 16.4 *
Scars, POD 60	59.4 ± 0.6	66.2 ± 0.5	35.4 ± 11.8 *
Scars, POD 90	58.8 ± 0.3	66.1 ± 0.5	14.9 ± 3.3
Scars, POD 120	58.9 ± 0.5	66.1 ± 0.3	15.5 ± 6.0

## Data Availability

The relevant data generated and (or) analyzed in the current study is available from the corresponding author upon reasonable request.
